# Three-dimensional imaging of mitochondrial cristae complexity using cryo-soft X-ray tomography

**DOI:** 10.1038/s41598-020-78150-3

**Published:** 2020-12-03

**Authors:** Carla C. Polo, Miriam H. Fonseca-Alaniz, Jian-Hua Chen, Axel Ekman, Gerry McDermott, Florian Meneau, José E. Krieger, Ayumi A. Miyakawa

**Affiliations:** 1 Brazilian Synchrotron Light Laboratory (LNLS), Brazilian Centre for Research in Energy and Materials (CNPEM), Campinas, SP 13083-970 Brazil; 2 Laboratory of Genetics and Molecular Cardiology, Heart Institute (InCor), University of São Paulo Medical School, São Paulo, SP Brazil; 3 Molecular Biophysics and Integrated Bioimaging Division, Lawrence Berkeley National Laboratory, Berkeley, CA USA; 4 Department of Anatomy, University of California San Francisco, San Francisco, CA 94158 USA

**Keywords:** Mitochondria, 3-D reconstruction, X-ray tomography

## Abstract

Mitochondria are dynamic organelles that change morphology to adapt to cellular energetic demands under both physiological and stress conditions. Cardiomyopathies and neuronal disorders are associated with structure-related dysfunction in mitochondria, but three-dimensional characterizations of the organelles are still lacking. In this study, we combined high-resolution imaging and 3D electron density information provided by cryo-soft X-ray tomography to characterize mitochondria cristae morphology isolated from murine. Using the linear attenuation coefficient, the mitochondria were identified (0.247 ± 0.04 µm^−1^) presenting average dimensions of 0.90 ± 0.20 µm in length and 0.63 ± 0.12 µm in width. The internal mitochondria structure was successfully identified by reaching up the limit of spatial resolution of 35 nm. The internal mitochondrial membranes invagination (cristae) complexity was calculated by the mitochondrial complexity index (MCI) providing quantitative and morphological information of mitochondria larger than 0.90 mm in length. The segmentation to visualize the cristae invaginations into the mitochondrial matrix was possible in mitochondria with MCI ≥ 7. Altogether, we demonstrated that the MCI is a valuable quantitative morphological parameter to evaluate cristae modelling and can be applied to compare healthy and disease state associated to mitochondria morphology.

## Introduction

Mitochondria are important intracellular specialized organelles present in all cell types, with the exception of red blood cells, critical for the generation of energy that fuels physiological process. Mitochondria metabolize biomolecules to generate energy in the form of ATP via the process of oxidative phosphorylation. Beyond this central role, mitochondria also produce metabolic precursors for macromolecules such as lipids, proteins, DNA and RNA. They also contribute to the maintenance of calcium homeostasis and activation of apoptosis, and generate metabolic by-products, such as reactive oxygen species (ROS) and ammonia^1^.

Ultrastructural analysis of mitochondria using electron microscopy revealed the presence of outer and inner mitochondrial membranes (OMM and IMM, respectively), which border the intermembrane space (IMS) and the matrix. The OMM is involved in mitochondrial lipid transport, intrinsic apoptotic pathway and mitochondrial fission and fusion processes^2^. The IMM consists of distinct morphological regions including the membrane boundary and the invaginations known as cristae, which are connected via cristae junctions. The cristae are the invagination of different shape and size of the IMM that significantly increase the surface area and harbor components required for oxidative phosphorylation (OXPHOS) and ATP production^3^. Cristae morphology determines assembly and stability of respiratory chain super-complexes and hence determine mitochondrial respiratory efficiency during cell life^4^. Cristae also sequester the bulk of cytochrome c molecules. During apoptosis, the curvature of the cristae membrane is inverted for the complete release of cytochrome c into the cytosol^5,6^.

Mitochondrial morphology is critical for cell function and survival. It can adapt to the cellular energetic state under both physiological and stress conditions by remodeling their morphology. For example, high energy demand increases the surface area of the cristae, while low energy demand leads to an expanded matrix and low cristae content^7^. Mitochondria can also change their shape, size and location through mitochondrial fusion and fission processes to provide appropriate distribution of mitochondria within the cells^8,9^. Thus, understanding the relationship of chemical composition to organelle morphologies and function, are essential for understanding biochemical processes in healthy and diseased cells^10^. It has been well established that structure-related dysfunction in mitochondria can lead to age-associated diseases in cardiopathies^11^ and neuronal disorders^12^.

Mitochondria and their internal structures are challenging to image using optical microscopy due their small size and their dynamic and sensitive behavior towards the many stresses inherent in living cells^13^. Optical fluorescence based microscopies, such as STED in living cells reached 50 nm resolution^14^ while PALM^15^ and STORM reached molecular-scale resolution of 20 nm^16^. The transmission electron microscopy (TEM) combined with tomography, allowed to extract information about a few microns in the mitochondria depth^17^. However, the organization of OMM and IMM inside a cell cannot be fully observed within the narrow depth of few microns provided by electrons. The light and electron-based techniques can provide high spatial resolution, but the volumetric information extracted is poor, due to the low penetration depth. The latter techniques also frequently require labeling and/or fixation procedures in sample preparation, which can induce to artifacts. On the other hand, the use of X-ray based imaging can provide information about thick and large area biological samples and under cryogenic conditions to avoid the radiation damage, for instance the modalities of X- ptychography of vegetal tissues^18^ and soft X-rays of whole cells^19^. Mitochondria has been already explored by X-ray imaging to be identified in the cellular context of wild-type single cells^19,20^, in cancer cells^21,22^ and isolated from the cells aiming to unveil its internal structure^23^. However, there are no reports of X-ray imaging-based analysis focusing on extracting 3D morphological and quantitative information about the OMM and IMM organization.

Soft X-ray tomography (SXT) emerged in the 90´s allowing to image whole cells without staining and chemical fixation^24^. The soft X-rays have higher penetration power in hydrated specimens as compared to electrons and produce bright-field images with higher resolution than light. The specimen illumination in the so-called “water window” of the electromagnetic spectrum (284–543 eV ) leads to specific elemental absorption lines in which organic material has ten times more contrast than water^25^. In this spectral region, the carbon and nitrogen main components attenuate the light transmission with an order of magnitude greater than does water^26,27^. This attenuation follows the Beer-Lambert law, being linear with thickness and a function of the biomolecular species present at each point in the specimen^28^. Consequently, it is possible to image thick and whole cells and to resolve intracellular structures, without staining agents^27,29^. Moreover, imaging specimens, in cryogenic conditions, with soft X-ray microscopy enables to maintain the specimens in their natural state and minimizes radiation damage during the data collection^30^.

In this work, we present a novel strategy for studying the ultrastructure of isolated mitochondria using cryo-soft X-ray tomography (SXT). Mitochondria structure were identified by the data analysis based on the 3D electron density provided by linear attenuation coefficient (LAC)^27^. The volume fraction and surface area of cristae and mitochondrial matrix compartments were accurately determined, and the cristae modelling was given by mitochondrial complexity index (MCI)^31^. It provided a quantitative parameter to characterize mitochondrial morphology which can be applied to reveal the dissimilarities between health and diseased mitochondria.

## Results and discussion

### Mitochondria identification by LAC in SXT images

When analyzing images from X-ray imaging, it is important to verify if radiation dose is causing sample damage^27^. The cryo-preservation is a fundamental step since it increases the radiation dose which the sample can withstand. Imaging the specimens at a cryogenic temperature at 110 K prevents radiation damage for doses of up to 10^10^ Gy^32^. The total adsorbed dose in our experiments was 4.57 × 10^7^ Gy, calculated according to Eq. 1 (Methods session) and considering mitochondria mass density and the experimental conditions. This dose is similar or lower compared to previous cryo- SXT dose values reported in the literature^24,29^. Moreover, we did not find compromised integrity of the structures or sample changes during the tomographic acquisition that could hamper further analysis.

Depending on the micro zone plate (MZP) used as objective we obtained 60 nm and 35 nm resolution^19^ data, which allowed us to identify mitochondria by observing the high-density cristae. The image contrast was enhanced after considering the effect of the point spread function (PSF) of the optics^33^ generating sharp edges and better defined mitochondrial cristae when compared with the non-linear reconstructions (Supplementary Fig. 1). Thus, the PSF reconstructions had a great importance to ensure accurate and realist measurements.

**Figure 1:**
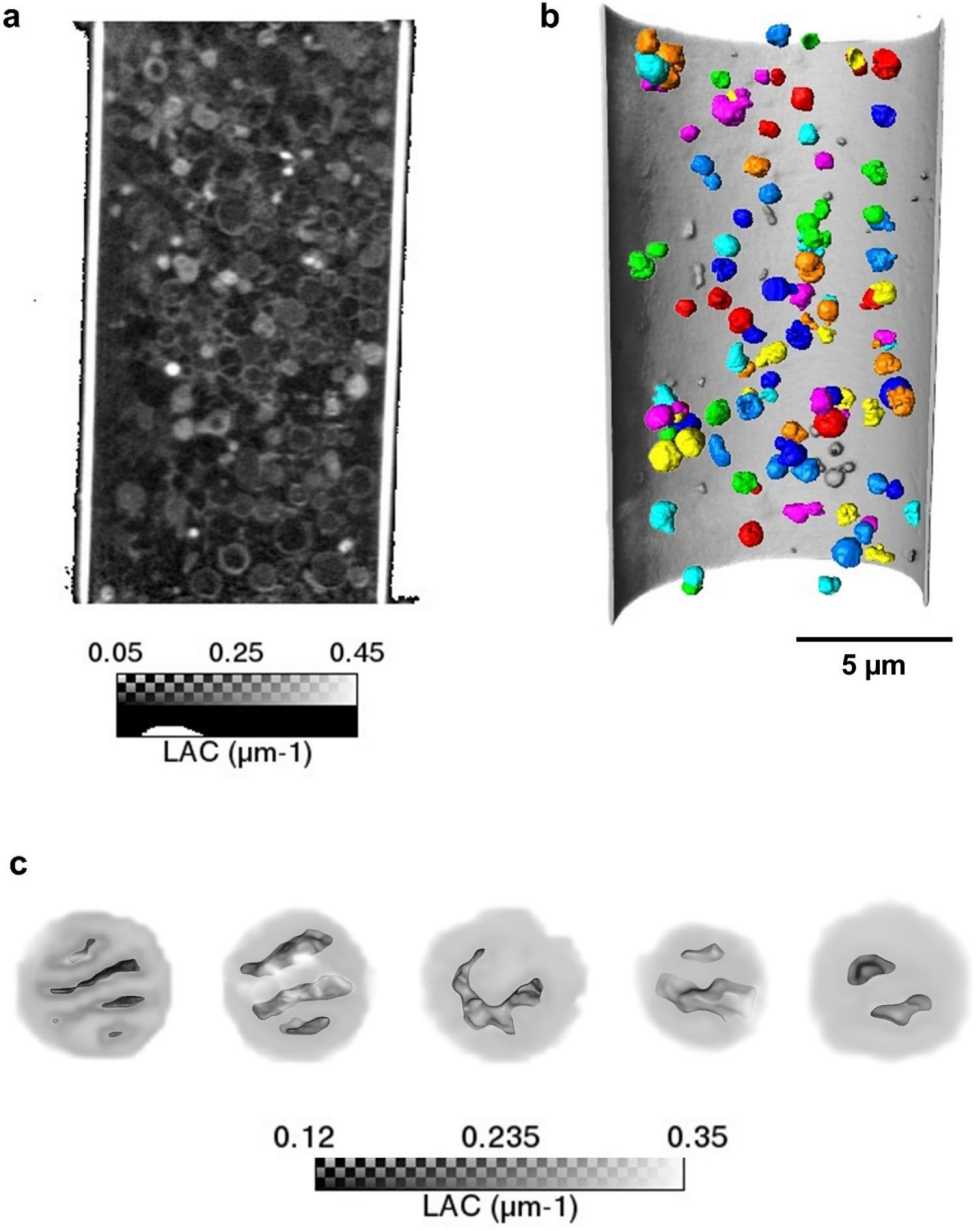
Image converted to linear coefficient attenuation (LAC) map. (**a**) Tomogram x,y slice, showing the macromolecules content within the capillary after the isolation procedure. The gray level corresponds to the LAC. (**b**) Image rendering highlighting the mitochondria which were extracted based on their LAC value to be further analyzed (**c**) rendering of structures identified as mitochondria according to the LAC and the morphological features of cristae and mitochondrial matrix.

Since the absorption of X-rays follows the Beer-Lambert’s law, each voxel contains quantitative information about attenuation allowing us to calculate the linear attenuation coefficient (LAC) (µm^-1^) for the pixel size and distinguish the different structures contained in the glass capillary. Although the mitochondria isolation process resulted in contamination with other intracellular structures (Fig. 1a), the LAC and the high resolution obtained with the cryo- SXT allowed to identify and to perform quantitative analysis with the mitochondria within the whole capillary.

**Figure 2:**
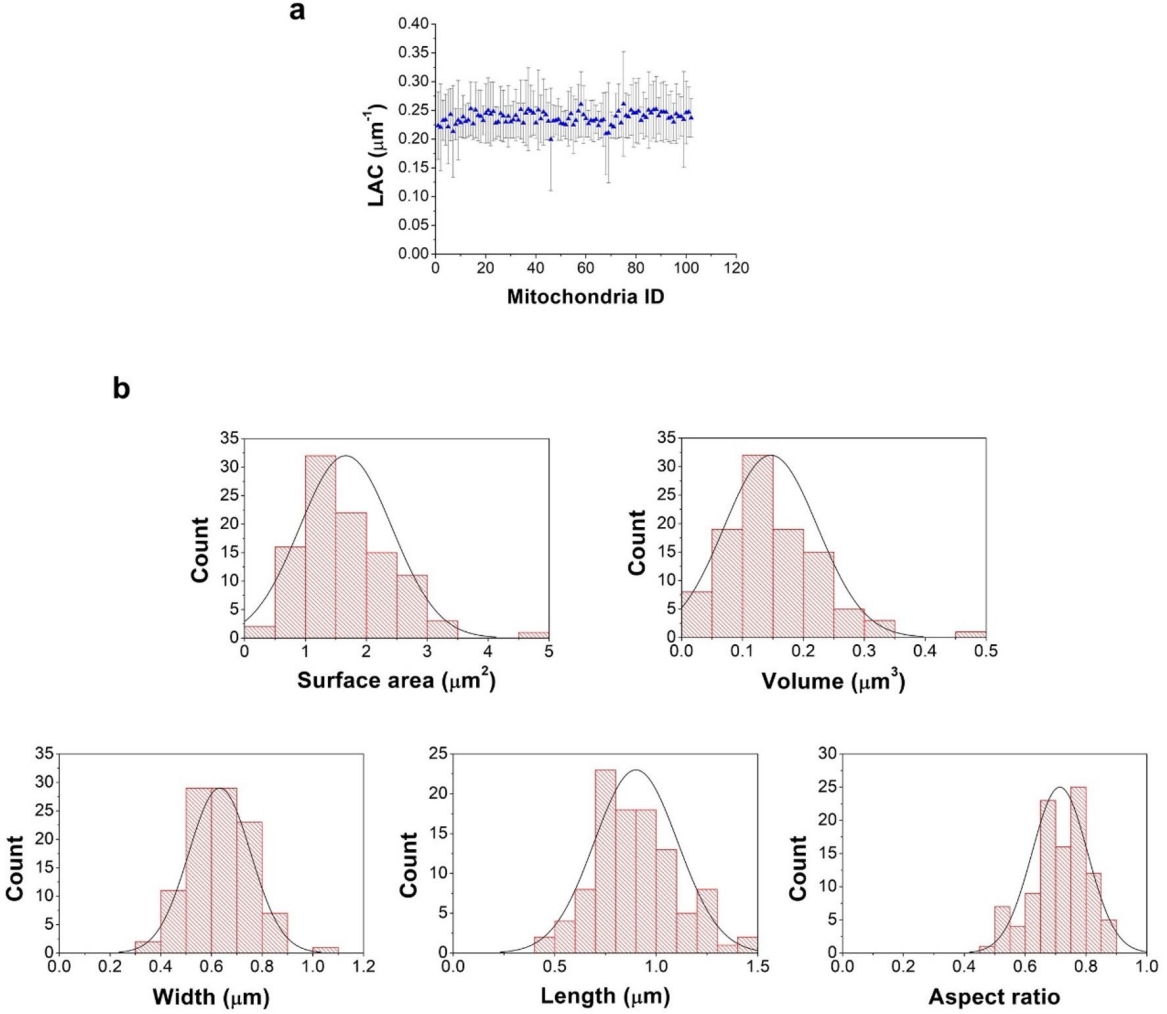
3D parameters distribution calculated for the analyzed mitochondria. (**a**) Average LAC calculated for each mitochondrion. (**b**) Geometric distribution found in the mitochondrial population and the normal fit.

We calculated the 3D LAC for the 102 mitochondria (Fig. 2a). The average LAC for the mitochondria contained in the capillary was 0.247 ± 0.04 µm^−1^. The 3D geometrical parameters for isolated mitochondria (Fig. 2b) was calculated showing surface areas of 1.66 ± 0.75 µm^2^, volumes of 0.11 ± 0.07 µm^3^, lengths of 0.90 ± 0.2 µm and widths of 0.63 ± 0.12 µm. Despite of the mitochondria population heterogeneity in terms of shape, the aspect ratio presented a normal distribution with peak at 0.7, showing a tendency to spherical morphology.

In the spectral region, known as the “water window”, between K-edges of carbon (E = 284 eV, λ = 4.4 nm) and oxygen (E = 543 eV, λ = 2.34 nm), water is relatively transparent whereas carbon rich molecules are one order of magnitude more strongly absorbing^25^. The reconstructed volume of the imaged samples in SXT results in a 3D density that describes the local LAC of the sample and indicates if the chemical composition of the structure provides sufficient contrast to identify biological features without any label^27^. Cell structures with a high water content, for example vacuoles, will be less absorbing and, consequently, will present less contrast than the higher carbon content organelles, such as lipid droplets^34^. The mitochondria LAC values are usually in between these two extremes along with other organelles such as vacuoles (0.20 µm^−1^) and heterochromatin (0.25 µm^−1^)^20,34–36^ and can represent a challenge to be distinguished. In the literature, it is described values of mitochondria LAC of 0.45 ± 0.03 µm^-1^ for *C. albicans*^35^; 0.36 ± 0.02 µm^−1^ for *S. cerevisiae*^34^; and 0.35 ± 0.03 µm^−1^ and 0.33 ± 0.03 µm^−1^ for *C. utilis*^20^. Here, we obtained mitochondrial LAC value of 0.247 ± 0.04 µm^−1^ and this lower value can be explained by the low carbon content of PBS, medium where the isolated mitochondria were resuspended. The cytoplasm is rich in biomolecules and consequently more strongly absorbing and gives higher values of LAC for mitochondria measured inside the cell.

Therewith, we presented a focused study on isolated mitochondria population with statistical analysis of geometry and LAC, unveiling the macromolecule 3D morphology.

### Morphological and quantitative characterization of features related to mitochondria structure

We selected 5 mitochondria (M1 to M5) from the capillary pool, identified based on LAC, where the resolution achieved allowed the observation of the internal structures (Fig. 1c).

**Figure 3:**
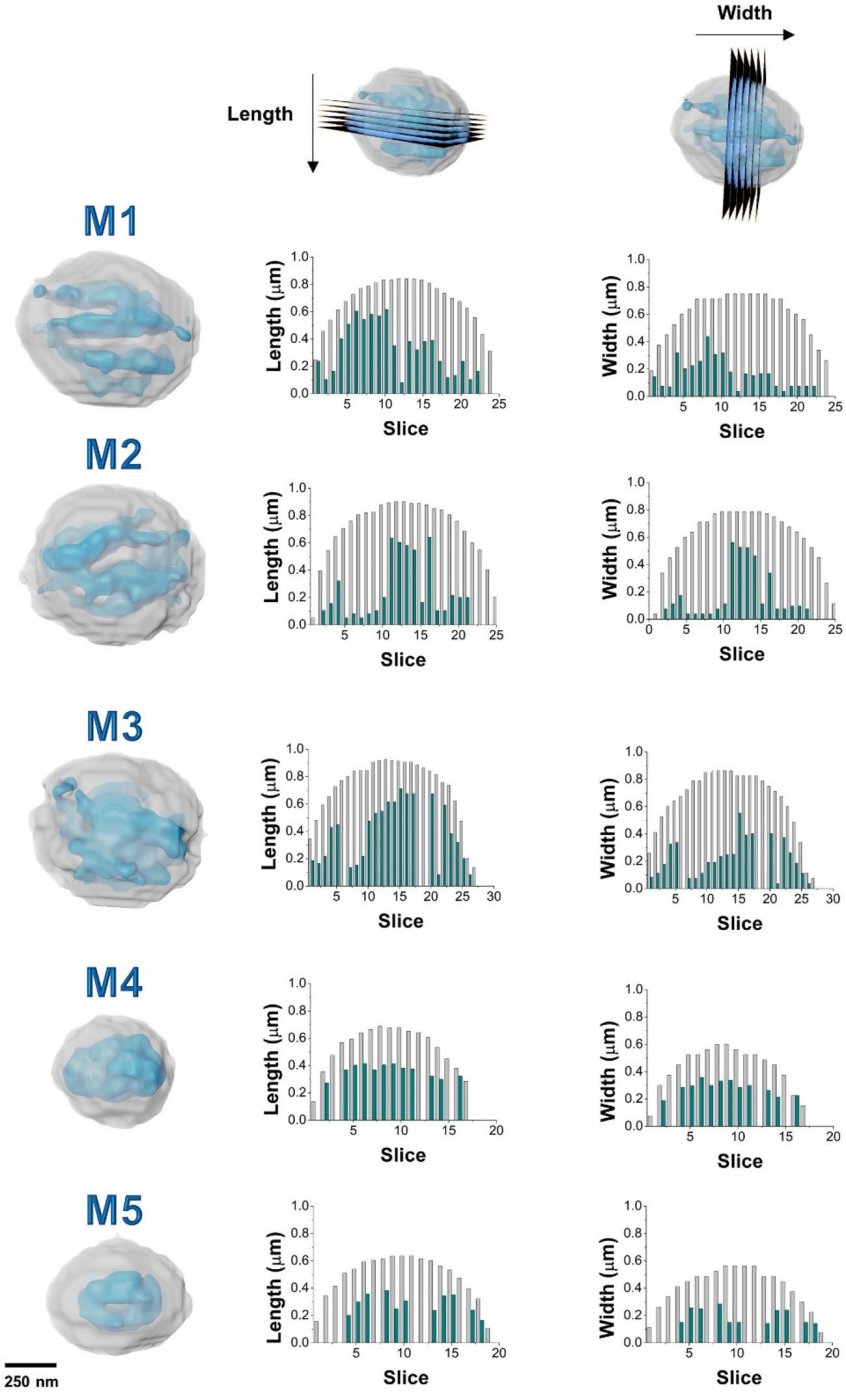
Mitochondria overall structure and compartmentalization. M1 to M5 renderings with the two compartments represented: cristae (gray and transparent) and the matrix (blue). All of them have spherical shape in accordance with the length and width calculated. A virtual slicing was performed in length direction and width direction and the measurement of each compartment was graphically represented with cristae in gray and matrix in blue.

Machine learning combined with watershed algorithm resulted in the separation between what here we call the cristae (comprising the OMM, IMM and IMS) and mitochondrial matrix (Fig. 3). As the resolution did not allow us to distinguish the separated OMM, IMM and IMS, we named the compartment containing those 3 structures as cristae as it can gives the notion of cristae configuration. The cristae correspond to 79 ± 0.04% of the total volume, while the matrix corresponds to 21 ± 0.04% (Table 1). Despite of the difference in M1 to M5 total volumes, the cristae to matrix ratio is maintained.

**Table 1:** Quantitative parameters extracted from mitochondrial cristae and matrix.

	Length (µm)	Width (µm)	Volume (µm^3^)			Volume fraction (%)		Surface área (µm^2^)		MCI
	Total	Total	Total	Cristae	Matrix	Cristae	Matrix	Cristae	Matrix	
M1	0.94	0.79	0.28	0.22	0.06	76	24	3.80	1.62	7.48
M2	0.96	0.84	0.30	0.25	0.05	79	21	3.52	1.15	7.17
M3	1.03	0.90	0.36	0.28	0.08	74	26	4.32	1.69	10.61
M4	0.72	0.62	0.11	0.09	0.02	78	22	1.67	0.50	5.35
M5	0.71	0.60	0.11	0.10	0.01	86	14	1.57	0.37	3.82

The matrix-to-cristae ratio distribution was compared using virtual slicing of length and width parameters (Fig. 3), indicating that, at this sampling, there is no pattern that could be established between these two compartments.

The scope of this study was to unveil the 3D mitochondrial cristae morphology using SXT to extract quantitative information. Thus, we applied the MCI modelling (Eq. 2, Methods) as previously applied to quantify the morphological complexity of smooth muscle mitochondria branches^31^. Cristae complexity was evaluated considering the parameters of the matrix surface area and volume to be sensitive to internal features. As more complex and higher area are generated by the invaginations, the more complex the structure will be and higher values for MCI will be obtained. Note that segmentation of mitochondria with MCI ≥ 7 (M1, M2 and M3 in Table 1) allowed a better visualization of the cristae into the mitochondrial matrix than mitochondria with lowers values of MCI (M4 and M5). As M4 and M5 have smaller dimensions (length and width), their structure could not be determined as the measurements were performed at the limits of resolution of the SXT. This data highlights the role of 3D high-resolution imaging to improve the structural and quantitative knowledge of important biomolecules such as the mitochondria.

Our study was performed using isolated murine mitochondria. It is known that mitochondria morphology depends on cytoskeleton organization and it may change if the cell is attached or in suspension. The same argument is valid for isolated mitochondria and it needs to be taken in consideration when studying mitochondrial structures. In most forms of microscopy, depth-of-field is linked to spatial resolution. The higher the spatial resolution the smaller depth of field^37^. In the SXT set up, the use of high spatial resolution optics (35 nm) results in a depth of field limited to imaging cells that are a few microns thick, for example yeasts, protozoan and bacteria. In our work, the mitochondria were isolated from vascular smooth muscles cells which are typically 100 µm in size and 5 mm maximum thick (determined by atomic force microscopy) limiting the possibility of imaging the mitochondria in the adherent culture. Thus, considering the current X-ray microscopes available, imaging and quantifying mammalian organelles is more straightforward if they are extracted from their cellular environment. The other advantage to using isolated organelles is the capability to collect statistically significant information from a single tomogram. The extracted organelles used in this study are derived from several cells and artifacts are excluded by collecting information of different mitochondria in a single sample. High resolution microscopy, such as X-ray based techniques, are time consuming and scientists depend on the short experiment times granted at external synchrotron facilities.

## Conclusion

In summary, we demonstrated that SXT 3D density information combined with the high-resolution optics is a powerful methodology to distinguish morphological features and extract accurate quantitative information about the mitochondrial architecture. The tens nanometers resolution imaging and the 3D density mapping based on the LAC of all the molecules present in the sample, allowed us to characterize mitochondria structure by soft X-ray microscopy. MCI modeling allowed us to characterize the morphology and to quantify the mitochondrial cristae complexity considering 3D parameters and providing cristae invagination structure. We envision that this methodology can be used to better understand cellular process associated to mitochondrial structure morphology such as apoptosis in cardiac and neurodegenerative diseases.

## Methods

### Cell culture

Smooth muscle cells were extracted from rat aorta using the explant protocol. Briefly, the endothelial cells were removed by mechanical friction and small fragments of vessels were placed in 6-well plate coated with gelatin. The fragments were let to adhere and maintained in Dulbecco’s modified Eagle’s medium supplemented with 20% FBS, 100U/mL penicillin and 100 μg/mL streptomycin. The cells derived from the explant were isolated and cultured up to 6th passage. The present study protocol was approved by the Institutional Review Board of the University of São Paulo Medical School, Brazil (SDS 3299/09/050, CAPPesq 0824/09), which follows the guidelines of the National Council of Ethics in Research, resolution 466. The methodology was carried out in accordance with relevant guidelines and regulations.

### Mitochondria extraction for cryogenic preparation

Smooth muscle cells cultured in T150 flask were harvested using trypsin and the pellet were homogenized in ice-cold mitochondria isolation buffer (210 mM mannitol, 70 mM sucrose, 1 mM EGTA, 0.5% albumin bovine serum, and 5 mM HEPES, pH 7.2). The cells were lysed in a 5 mL tissue grinder and the homogenate was centrifuged at 700×*g* for 8 min at 4 °C. The resulting supernatant was centrifuged at 17,000*g* for 15 min at 4 °C and the mitochondrial pellet was resuspended in a minimal amount of phosphate buffer solution (PBS). The efficiency of mitochondria preparation was verified by positive staining of specific markers: voltage-dependent anion channel 1 (VDAC1) and optic atrophy type 1 (OPA1) (Supplementary Method Fig. 1). Custom designed capillaries were manually pulled to about 5 µm diameter tips and 1–2 µL of mitochondria solution was loaded using a microloader. The capillaries were plunge-frozen in liquid propane at 90 K using an in house developed apparatus to ensure the sample vitrification.

### Cryogenic soft X-rays tomography (cryo-SXT)

Capillaries containing the cryo-preserved mitochondria were place in the soft X-ray microscope^38^ of XM-2 beamline in the National Center for X-ray Tomography (http://ncxt.lbl.gov) at the Advanced Light Source (http://www.als.lbl.gov) of Lawrence Berkeley National Laboratory (Berkeley, CA). The experiments were carried out in ‘water window’ illumination (517 eV) using either a 60-nm or 35-nm Fresnel zone plate. This optic defined the maximum spatial resolution of the tomographic reconstruction. Samples were kept in a stream of liquid-nitrogen-cooled helium gas during transferring and data collection to avoid thawing up and radiation damage^35,38^. For each data set, 90 projection images were collected sequentially in rotation axis with 2° increments, with a total rotation of 180°, using exposure times from 200 to 400 ms, depending on the sample thickness.

The deposited energy mass due to absorption, *D* (Gy), was calculated:

1 $$ D = \frac{F \mu EEt}{\rho} $$

where *F* is the photon flux in ph/m^2^/s, *µ* is the calculated linear coefficient attenuation for mitochondria, considering the formula H_50_C_30_N_9_O_10_S_1_, *E* is the energy in kg m^2^/s^2^, *Et* is the total exposure time and *ρ* is the mass density.

The software suite AREC3D was used to align the projection images calculate tomographic reconstructions^39^ followed by linear point spread function (PSF) approximation reconstruction^33^.

### Image analysis

The linear attenuation coefficient (LAC) was calculated and applied for the whole images^27^. The LAC was used to identify the mitochondria and separate them from other intracellular structures present in the buffer solution. The images segmentation was initially performed using the Trainable Weka Segmentation (TWS) machine learning tool^40^ available in Fiji^41^ to separate the different cellular compartments. In TWS, the 3D input images were submitted to pixel classification where a set of input pixels were manually selected and attributed to a specific class, either in cristae or mitochondrial matrix. The selected classified pixels, i.e. the classifiers, were used to automatically classify the rest of the pixels of the complete images, based on grey value level, texture, morphology, among other features. The program output was two separate grey level images for cristae and mitochondrial matrix. The crista image was processed using the watershed algorithm to generate two labels (8-bit binary file) from the cristae space and from its interior, which corresponds to the mitochondrial matrix. The calculations of surface area, volume fraction and 3D measurements were performed using the specific modules of Avizo. We calculated the mitochondrial matrix shape using the mitochondrial complexity index (MCI) which is a 3D modeling analogous to sphericity and scales with mitochondrial shape complexity^31^:

2 $$ {\text{MCI}} = \frac{{{\text{SA}}_{{\text{m}}}^{{3}} }}{{{\text{16}}\uppi^{{2}} {\text{V}}_{{\text{m}}}^{{2}} }} $$

where SA_m_ is the mitochondrial matrix surface area and V_m_, the total mitochondrial matrix volume.

## Supplementary information

Supplementary Information.

## Data Availability

The data reported in this article are available from the corresponding authors upon reasonable request.
