# Supplementary material for: Three-dimensional imaging of mitochondrial cristae complexity using cryo-soft X-ray tomography
Source: Sci Rep. 2020 Dec 3;10:21045. doi: 10.1038/s41598-020-78150-3 (PMC7713364; doi:10.1038/s41598-020-78150-3)
Supplement: Supplementary file 1 — Supplementary Information. [file 41598_2020_78150_MOESM1_ESM.docx]

**Supplementary information**

**Three-dimensional imaging of mitochondrial cristae complexity using cryo-soft X-ray tomography**

Carla C. Polo^1*^, Miriam H. Fonseca-Alaniz^2^, Jian-Hua Chen^3,4^, Axel Ekman^3^, Gerry McDermott^3^ Florian Meneau^1^, José E. Krieger^2^, Ayumi A. Miyakawa^2*^

^1^Brazilian Synchrotron Light Laboratory (LNLS), Brazilian Center for Research in Energy and Materials (CNPEM), 13083-970, Campinas, SP, Brazil

^2^Laboratory of Genetics and Molecular Cardiology, Heart Institute (InCor), University of São Paulo Medical School, São Paulo, SP, Brazil

^3^Molecular Biophysics and Integrated Bioimaging Division, Lawrence Berkeley National Laboratory, Berkeley, California, USA

^4^Department of Anatomy, University of California San Francisco, San Francisco, California, 94158, USA

*e-mail: [carla.polo@lnls.br](mailto:carla.polo@lnls.br) and [ayumi.miyakawa@incor.usp.br](mailto:ayumi.miyakawa@incor.usp.br)


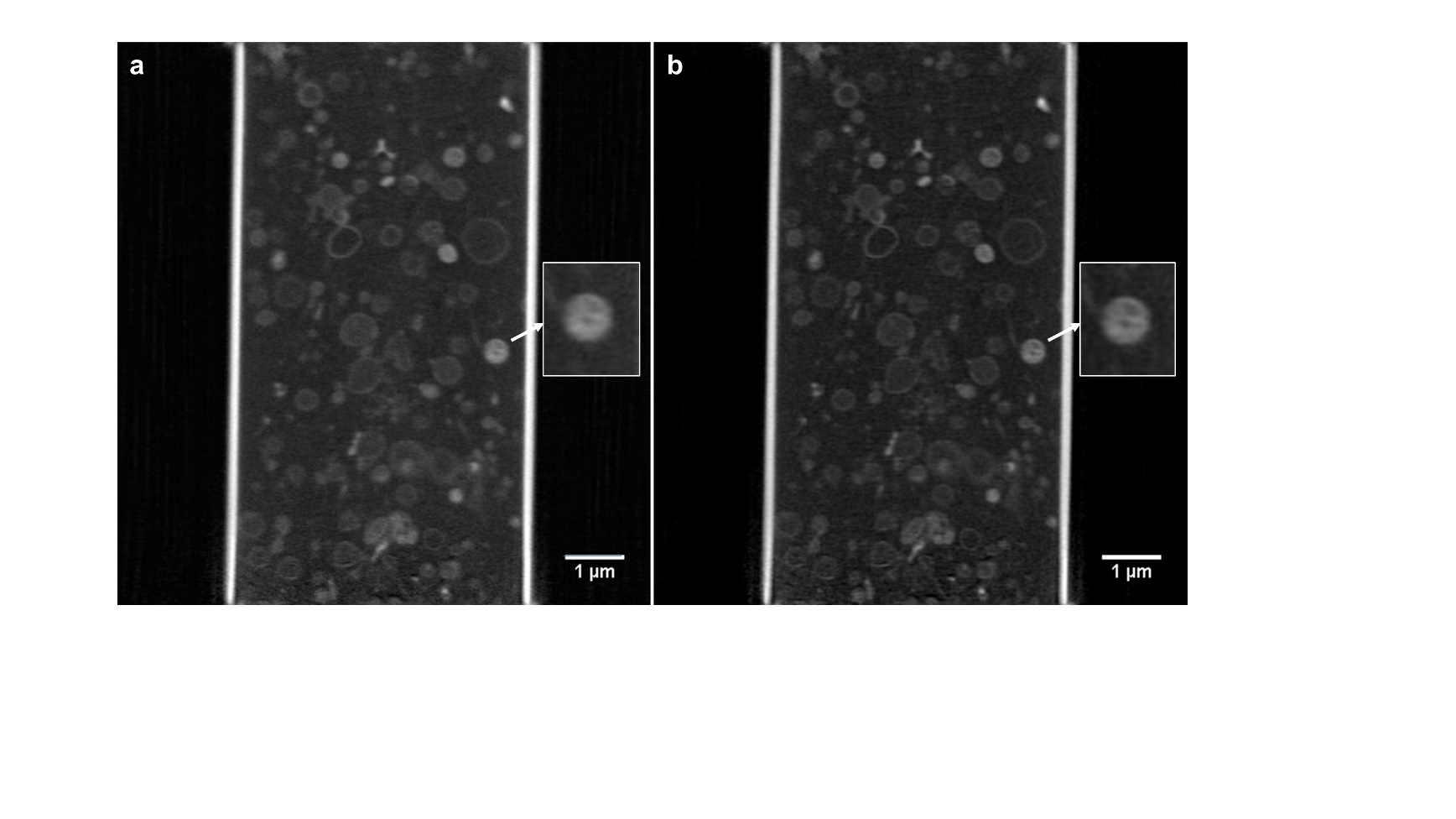


**Supplementary Fig. 1**. **SXT reconstructed images**. The glass capillaries contain the extracted organelles embedded in amorphous ice. (**a**) Image reconstructed with the non-linear deconvolution approach AREC package^28^. (**b**) Reconstruction using linear approximation including the effect of the point spread function (PSF) of the optics. The contrast is increased in the PSF reconstruction, highlighting edge features as it is depicted for the mitochondrial cristae (the insets).


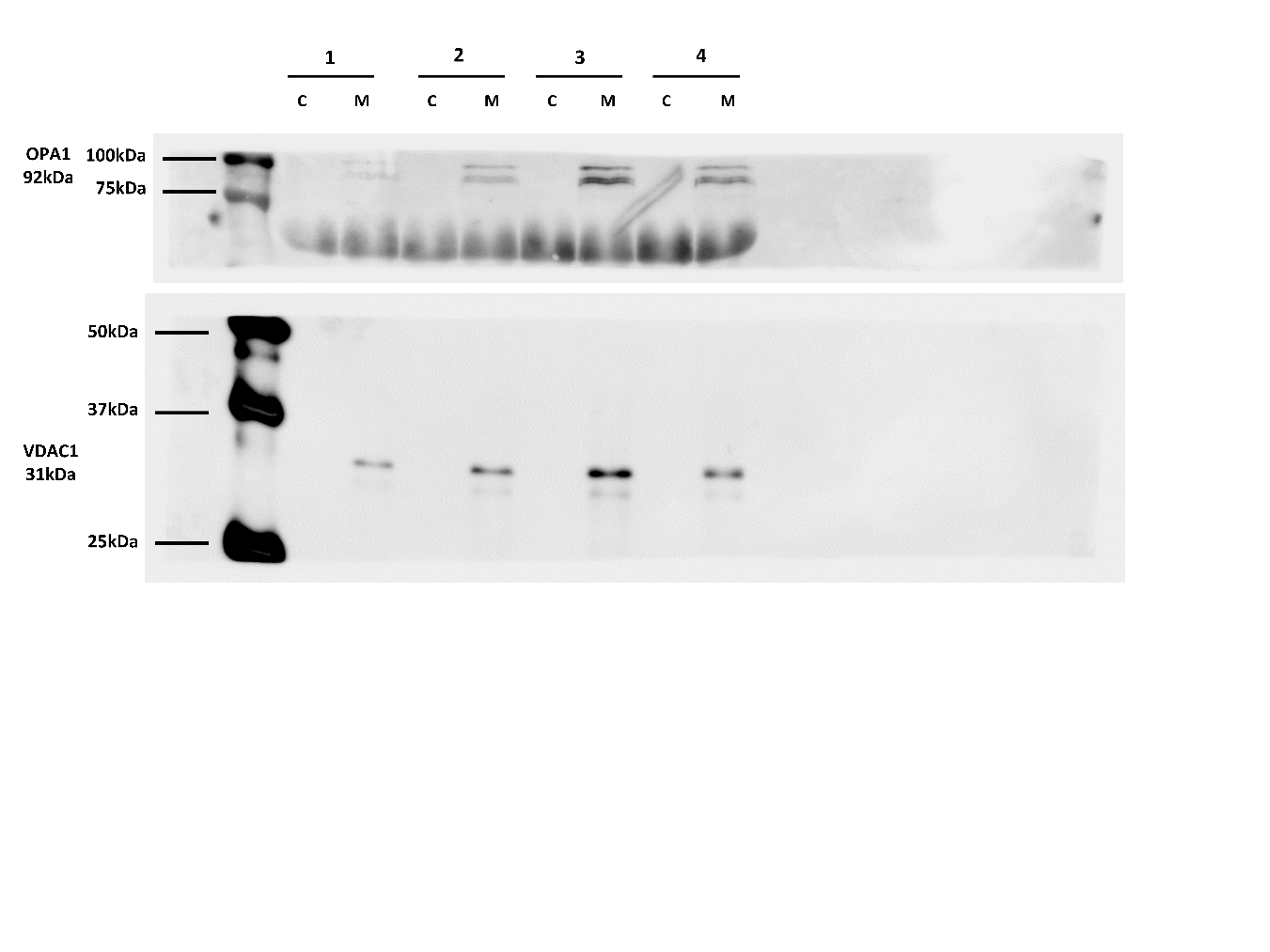


**Supplementary Method 1. Western blot analysis of cytosolic (C) and mitochondrial (M) fractions isolated from cultured rat aortic smooth muscle cells (rAo-SMC)**^1^**.** Western blots show enrichment of mitochondrial membrane proteins, voltage-dependent anion channel 1 (VDAC1) and optic atrophy type 1 (OPA1), in mitochondrial fraction as compared to cytosolic fraction in 4 different smooth muscle cells extraction.

# Supplementary reference

1 H. A. Ruiz, R. M. Rodríguez-Jasso, B. D. Fernandes, A. A. Vicente and J. A. Teixeira, *Renewable and Sustainable Energy Reviews*, 2013, **21**, 35–51.
